# Age as a determinant of nutritional status: A cross sectional study

**DOI:** 10.1186/1475-2891-4-28

**Published:** 2005-10-27

**Authors:** Sarah Forster, Salah Gariballa

**Affiliations:** 1 Sheffield Institute for Studies on Ageing, The University of Sheffield & Barnsley Hospital, Northern General Hospital, Sheffield, S5 7AU, UK

## Abstract

**Background:**

Undenutrition is known to be prevalent and largely unrecognised in older patients; however, aberrations in indicators of nutritional status may simply reflect effects of age and/or functional disability.

**Objective:**

The aim of this study was to measure the effect, if any of age on nutritional status in older patients.

**Design:**

445 randomly selected hospitalised patients consented to nutritional status assessment derived from anthropometric, haematological, and biochemical data within 72 hours of admission. Nutritional status was compared between those age < 75 years and those aged 75 years or more. Using multiple regression models, we measured the association between age and nutritional assessment variables after adjusting for disability, chronic illness, medications, smoking and tissue inflammation.

**Results:**

Body weight, body mass index, mid-upper arm circumference, haemoglobin, serum albumin and plasma ascorbic acid were all significantly lower in people aged ≥ 75 years compared with those < 75 years of age. Although riboflavin (vitamin B2), 25OH VitD_3_, red-cell folate and vitamin B_12 _concentrations were lower in those aged ≥ 75 years, differences were not statistically significant. After adjusting for disability and co-morbidity in a multivariate analysis, age alone had a significant and independent effect on important anthropometric and biochemical nutritional assessment variables.

**Conclusion:**

Increasing age is independently associated with poor nutritional status. This may partly explain the poor clinical outcome in older patients.

## Introduction

Societies worldwide have experienced a considerable increase in the number of older people [1]. There is a growing recognition that age-related physiological changes may predispose to protein-energy undernutrition, in the elderly, particularly in the presence of other factors associated with aging, including social and psychological, variables and the presence of disease [2,3]. Consequently, undenutrition is known to be prevalent and largely unrecognised in older patients. There is also evidence which links protein-energy undernutrition or its markers with clinical outcomes in acute and non-acute hospital settings, and additional evidence indicating that nutritional supplements can improve outcome in some of these settings [4-6].

Good nutrition may contribute significantly to the health and well being of older individuals, and to their ability to recover from illness. However there is no gold standard for diagnosing undernutrition, and most clinically available nutrition screening instruments lack sensitivity and specificity. In addition, abnormal nutritional indicators may simply reflect effects of age, functional disability, or severe underlying disease [7,8]. Furthermore, the difficulties in detecting early signs of undernutrition are similar to those encountered in the early recognition of many age-related diseases [7]. However, in the case of nutritional deficiency there are two further difficulties: for almost every nutrient there is a long latency period before a low intake leads to overt clinical manifestations, and early diagnosis must depend upon the findings of abnormalities of special tests, including biochemical and haematological investigations. Second, in the elderly the true significance of abnormal results of these tests is not fully understood [9]. The aim of this study was to measure the effects of aging on nutritional status in older patients.

## Subjects & Methods

Four hundred and forty five acutely ill older patients who took part in a randomised, double blind, placebo-controlled single-centre trial of nutritional supplementation were included. The randomisation sequence was generated by the trial statistician, concealed in sequentially numbered, sealed opaque envelopes and kept in a clerical office at a different city. Admission diagnoses included ischaemic heart disease (myocardial infarction & angina), heart failure, atrial fibrillation, chronic obstructive pulmonary disease, chest and urinary tract infections, septicaemia, stroke, Parkinson's disease anaemia, diabetes, osteoarthritis, rheumatoid arthritis syncope, falls, fracture limbs, elective surgery knee and hip surgery. Inclusion criteria were: age ≥ 65 years; stable medical condition; able to swallow and able to sign an informed written consent form. Patients excluded from the study were those with severe medical or psychiatric illness including those with gastric surgery, malabsorption, or morbid obesity, in coma, diagnosed severe dementia (abbreviated mental test < 6), and malignancy, living in institution and patients already on supplements. The study was approved by Barnsley research ethics committee.

## Clinical and nutritional assessment

Following informed written consent and recruitment to the study all patients had baseline assessment. The assessment included demographic and medical data including current diagnosis, history of chronic illnesses, smoking, alcohol and drug intake, nutritional status and disability (Barthel score).

Nutritional status was assessed from anthropometric, haematological and biochemical data within 72 hours of admission. All anthropometrics measurements were performed by a single observer (SF) using standard methods with intra observer's differences assessed prior to the commencement of the study. Mid-upper arm circumference (MUAC) and triceps skin folds (TSF) were measured by a flexible tape and Harpenden Skin fold callipers accurate to 0.2 mm (Practical Metrology Sussex UK) respectively and the mean of three measures was recorded. The local Pathology Laboratory performed routine tests including serum ferritin, albumin and transferrin measurements. C-reactive protein (CRP) concentration, a marker of tissue inflammation, was measured by a modified latex-enhanced immuno-turbidimetric assay (normal ≤10 mg/L). The inter-assay coefficient of variation (c.v.) was 3.9%. Red cell folate and plasma vitamin B_12 _were measured on the Architect (Abbott Laboratories) using chemiluminescent microparticle immunoassay technology. The inter-assay coefficients of variation were 12.6% and 8.4% respectively. Riboflavin status was assessed as the erythrocyte glutathione reductase activity coefficient (EGRAC), using the Cobas BioAutoanalyser (Roche Diagnostics), giving an inter-batch cv of 7.6%. Ratios above 1.4 were considered to reflect biochemical deficiency of riboflavin [10]. Plasma total ascorbic acid was measured by a fluorescence assay automated for the Cobas BioAutoanalyser [11], giving inter-batch cv's of 8.4%. 25-Hydroxy Vitamin D3 was measured by HPLC using Chromsystems (Germany). This method uses a Chromsystems reagent kit, which allows the safe chromatographic determination of 25-OH-vitamin D3 on a simple isocratic HPLC system with UV detection [12]. The inter-assay coefficient of variation was 4.6%.

Disability at baseline was assessed using the Barthel score on a 20-point scale [13]. The Barthel scores 10 functions on a scale 0 (fully dependent) to 20 (independent).

## Statistical analyses

Statistical analyses were performed with SPSS software, version 11.0 (SPSS Inc., Chicago). Descriptive tests (median and inter-quartile range) were used to describe the baseline characteristics of the subjects. Mann Whitney U tests was used to test between-group differences where appropriate with a p-value of <0.05 regarded as statistically significant. A forward stepwise multiple regression analysis was performed to determine the influence of age on nutritional status as measured by body mass index (BMI), MUAC, TSF, haemoglobin, serum albumin and vitamin C after adjusting for a number of independent clinical variables including disability (Barthel score), co morbidity (previous illnesses, drugs and smoking) and tissue inflammation (CRP).

## Results

Between July 2001, and May 2004, 445 patients were recruited. Table 1 shows baseline clinical characteristics including gender, smoking, alcohol, consumption, chronic illness, drugs, disability, mental status, tissue inflammation (CRP), haemoglobin, glucose and renal function.

**Table 1:** Baseline characteristics of subjects age < 75 years compared with those aged 75 years or more [n(%) unless stated otherwise]

Variable		**<75 years (n = 181)**	**≥75 years (n = 264)**
**Gender, female**		78 (43)|	133 (50)
**Smoking**	Never smoked	50 (28)	87 (33)
	Ex smoker	80 (44)	136 (52)
	Current smoker	44 (24)	37 (14)
**Number of subjects with alcohol >14 units**		26 (14)	20 (8)
**Chronic disease**	IHD	58 (32)	104 (39)
	Hypertension	63 (35)	80 (30)
	Stroke	14 (8)	37 (14)
	COPD	35 (19)	49 (19)
**Medications +**	0–2	66 (38)	77 (30)
	3–6	97 (55)	166 (64)
	>6	12 (7)	16 (6)
Barthel Score *, median (Q1-Q3)		19 (14–20)	18 (13–20)
**C-reactive protein median (Q1-Q3)**		17 (5–79)	19.7 (5–59)
**Fasting blood glucose median (Q1-Q3)**		5.6 (5–6.4)	5.6 (5–6.2)

* P < 0.05

+ Values do not always add up because of some missing data

Subjects aged ≥75 years were significantly more disabled and had non significantly higher levels of co-morbidity and CRP concentration compared with those less than 75 years of age. Age alone affected both anthropometric and nutritional biochemical measurements. BMI, MUAC, haemoglobin, serum albumin and plasma ascorbic were all significantly lower in persons > 75 years of age compared with those younger than 75 years (Table 2). Riboflavin (EGRAC), 25OH VitD_3_, red cell folate and vitamin B_12 _concentrations were nonsignificantly lower in those aged ≥75 years.

**Table 2:** Anthropometric & biochemical nutritional data of subjects age < 75 years compared with those aged 75 years or more [median(interquartile range)]

	**N**	**<75 years old**	**N**	**≥75 years old**
**Body weight (kg)**	166	70.0 (60–79)	231	63.5 (56.5–72.5)**
**BMI + **	165	26 (23–28)	231	25 (22–27)*
**MUAC (cm) + **	175	29.5 (26.5–32.0)	259	27.5 (25.5–29.5)**
**TSF (mm) + **	174	16 (11–21)	259	15 (11–19)
**Haemoglobin (g/dl)**	181	13.3 (12–15)	259	12.8 (11–14) **
**Albumin (g/L)**	164	39 (36–42)	245	37 (34–40)**
**Transferrin (μg/L)**	143	2.15 (1.79–2.44)	199	2.10 (1.76–2.51)
**Ferritin (pmol/L)**	156	254 (103–497)	215	238 (124–521)
**Red cell folate (nmol/L)**	147	378 (437–888)	210	359 (269–504)
**Vitamin B_12 _(pmol/L)**	154	239 (170–349)	215	214 (154–337)
**Vitamin B_2 _(EGRAC)**	124	1.27 (1.18–1.40)	174	1.28 (1.18–1.38)
**Plasma ascorbic acid (μmol/L)**	129	21.3 (18.7–35.8)	193	15.2 (5.6–32.5)*
**25OH Vit D_3 _(nmol/L)**	107	36.0 (18.0–59.0)	145	33.0 (17.2–53.5)

** p value < 0.01, * p value < 0.05

+ body mass index (**BMI**), Mid-upper arm circumference (**MUAC**) and triceps skin folds (**TSF**)

Tables 3 &4 summarize results of the multiple regression analysis for the association between age and other clinical variable and nutritional assessment parameters. Among those included in the multiple regression models, age and smoking revealed a statistically significant association with BMI, MUAC and TSF (Table 3). However, haemoglobin and serum albumin were primarily associated with age, disability (Barthel score) and tissue inflammation. Vitamin C values were mainly predicted by smoking and tissue inflammation. For individuals, aged ≥ 75 years BMI, MUAC and TSF were 1.3, 1.95 cm and 0.02 mm less respectively compared with those patients aged less than 75 years (Table 3). Similarly for patients aged 75 years or more haemoglobin, serum albumin and vitamin C values were 0.4 g/dL, 1.5 g/L and 4.4 μmol/L less respectively, compared with patients aged less than 75 years. However the results for vitamin C did not reach statistical significance (Table 4).

**Table 3:** Multiple regression result of age, disability, co-morbidity, smoking and acute phase response on BMI, MUAC and TSF

	**Body mass index**		**MUAC (cm)**		**TSF (mm)**	
	**Regression coefficient (95% C.I)**	**P value**	**Regression coefficient (95% C.I)**	**P value**	**Regression coefficient (95% C.I)**	**P value**

**Age (<75; ≥ 75 yrs)**	-.1.30 (-2.0 to -.27)	**0.010**	-1.95 (-2.7 to -1.2)	**0.000**	-0.02 (-0.09 to-0.01)	**0.009**
**Barthel score (<11; ≥ 11)**	-.13. (-1.3 to 1.1)	0.832	0.43 (-0.5 to 1.3)	0.352	-0.03 (-0.5 to 1.3)	0.556
**Chronic illnesses**	.036 (-.20 to .41)	0.508	-0.02 (-0.2 to 0.3)	0.618	-0.05 (-0.01 to 0.02)	0.310
**All medications**	-.035 (-.32 to .16)	0.523	-0.14 (-0.3 to 0.1)	0.162	0.02 (-0.01 to 0.01)	0.772
**Smoking (Never, Ex, Current)**	-1.16 (-1.78 to -.55)	**0.000**	-0.87 (-1.4 to -0.4)	**0.001**	-0.02 (-0.08 to -0.02)	**0.000**
**CRP (≤10, >10 mg/L)**	.01 (-.94 to 1.7)	0.062	-0.02 (-0.7 to 0.8)	0.824	0.05 (-0.02 to 0.06)	0.305

**Table 4:** Multiple regression result of age, disability, co-morbidity, smoking and acute phase response on haemoglobin, Serum albumin and vitamin C

	**Haemoglobin **(g/dl)		**Serum Albumin **g/L		**Vitamin C μmol/L**	
	**Regression coefficient (95% C.I)**	**P value**	**Regression coefficient (95% C.I)**	**P value**	**Regression coefficient (95% C.I)**	**P value**

**Age (<75; ≥ 75 yrs)**	-0.4 (-0.9 to -0.03)	**0.035**	-1.5(-2.3 to -0.7)	**0.000**	-4.4 (-9.2 to .41)	0.073
**Barthel score (<11; ≥ 11)**	1.0 (0.5 to 1.6)	**0.000**	2.5 (1.5 to 3.5)	**0.000**	5.6 (-0.4 to 11.5)	0.066
**Chronic illnesses**	0.02 (-0.11 to 0.18)	0.650	0.2 (-0.1 to 0.5)	0.190	0.3 (-1.3 to 2.0)	0.709
**All medications**	-0.03 (-0.15 to 0.08)	0.575	0.04 (-0.1 to .3)	0.407	0.8 (-0.5 to 2.1)	0.225
**Smoking (Never, Ex, Current.)**	0.03 (-0.19 to 0.39)	0.514	-0.38 (-0.9 to 1.9)	0.191	-3.8 (-7.1 to -.53)	**0.023**
**CRP (≤10, >10 mg/L)**	-0.9 (-1.3 to -0.5)	**0.000**	-3.7 (-4.6 to -2.9)	**0.000**	-6.5 (-11.5 to -1.5)	**0.011**

## Discussion

The main findings of this study were that patients aged 75 years or more had poorer nutritional status compared with those younger than 75 years. After adjusting for nonnutritional clinical risk indicators, increasing age was strongly and independently correlated with poor nutritional status.

Ageing in man is accompanied by changes, which may impair food acquisition, digestion, and metabolism. Anorexia and weight loss are common and important clinical problems in older people, and the causes are multifactorial [2,3]. There is a growing recognition that age-related physiological anorexia may predispose to protein-energy undernutrition in older persons, particularly in the presence of other pathological factors associated with aging [2,3]. For example, alteration in smell and taste and poor dental health directly decrease food intake or influence food selection [7]. In general, physical activity and lean body mass decrease with aging, while body fat, increases. These factors may decrease energy requirements and intake [7]. Lower food intake may lead to lower intake of both macro- and macronutrients, and even in relatively healthy persons, mild, sub-clinical nutritional deficiencies are known to be common. For example, the most recent National Diet and Nutrition Survey people aged 65 years and older in the UK highlighted low dietary intakes and poor biochemical status for a range of micronutrients including vitamin C, riboflavin, thiamine and folate, and modest intakes of vitamin E [14].

In this study we also found a significant independent association between former and current smokers status and poor nutritional status. Smoking is known to be associated with oxidative stress, poor nutritional status, weight loss, and increased mortality [15,16]. Because smokers are known to have low intakes of fruits and vegetables that are rich in antioxidants, they are therefore more likely to be susceptible to oxidative damage caused by free radicals [15,16]. Palaniappan et al. have recently shown that smokers consume significantly fewer fruits and vegetables than non-smokers, leading to lower intake of folate and vitamin C [16]. Although mechanisms leading to poor nutritional in smokers are not known, possible contenders include poor taste and appetite and pro inflammatory effect of smoking or possible confounding effect.

Numerous studies in the past have demonstrated a strong association between serum albumin levels and clinical outcome [4,5,17]. A study of predictors of early non-elective hospital readmission in elderly patients has found that individuals with any amount of weight loss and no improvement in albumin concentrations during the first month after hospitalisation were at a much higher risk of readmission than were those who maintained or increased their post-discharge weight and had repleted their serum albumin concentrations [17].

Many conditions such as disability, acute and chronic diseases may influence nutritional status in ageing patients [4,18-20]. Because poor nutritional status may be partly related to clinical risk indicators other than age, such as underlying disease state, tissue inflammation, diet-medication interactions, or functional capacity, we made an attempt to control for these factors. By adjusting for the influence of these factors on nutritional status, it was possible to identify a potential independent effect of age on nutritional status. Because patients with diseases severe medical and psychiatric illnesses such as liver, gastrointestinal, kidney or neoplasm were excluded from this study, it is possible that increasing age alone is causally related to poor nutritional status.

Although in this cross-sectional study we have identified age as a potentially important risk factor for undernutrition in a hospital inpatient population, our results may not be extrapolated to healthy populations of older individuals.

It is also not clear whether nutritional deficiencies in individuals aged 75 years and older reflect poor nutrient intake, increased demand for nutrients, or simply represent underlying co-morbidities. Although this study was not designed to answer these questions, targeting this cohort with nutritional supplementation may help to overcome the potentially detrimental effects of nutritional deficits in this population.

Nutritional support studies to determine the optimal timing and composition of nutritional therapy relative to a patient's age are long overdue. This has the potential to improve nutritional status and lead to better rehabilitation outcome, decreased readmission rate, improved quality of life, and contribute to reducing health care costs.
